# Editorial Note: Clinical and Parasitological Protection in a *Leishmania infantum*-Macaque Model Vaccinated with Adenovirus and the Recombinant A2 Antigen

**DOI:** 10.1371/journal.pntd.0012499

**Published:** 2024-09-12

**Authors:** The PLOS Neglected Tropical Diseases Editors

Concerns have been raised that lanes 4 and 5 in Fig 3B of this article [1] appear highly similar.

In response, the authors stated that lanes 4 and 5 in Fig 3B may show the same data. The authors intended to show that lysates of host cells infected with an unrelated adenovirus 5 encoding the *T*. *cruzi* ASP2 antigen (lane 4) and a lysate of uninfected host cells (lane 5) did not react with a monoclonal antibody specific for the A2 protein. They stated that a positive reaction can be seen in lanes 2 (recombinant A2 protein) and 3 (recombinant adenovirus 5 expressing A2). The authors stated that the underlying data for this article [1] are no longer available but provided a western blot from an additional experiment with plasmids and adenovirus 5 encoding the A2 gene and related controls (S1 File). They also stated that Fig 3A in their previous work [3] shows expression of A2 protein by adenovirus 5-A2-infected or plasmid A2-transfected cell lysates, but not from cells infected with adenovirus ASP2, and Fig 1B in [4] shows the ectopic expression of ASP2 by adenovirus 5-infected host cells in a western blot revealed with a monoclonal antibody specific for ASP2 protein.

A member of the *PLOS Neglected Tropical Diseases* Editorial Board reviewed the concerns and authors’ responses, and advised that the data in S1 File satisfactorily demonstrate negative control results as needed to address the Fig 3B concerns.

While the similarities between lanes 4 and 5 in Fig 3B in [1] have not been fully resolved, we also concluded that the article’s overall results and conclusions are not significantly affected since they relate to negative controls and appear to be supported by other results.

During editorial follow-up, it was noted that several panels in Fig 7 in [1] appear similar to panels in Figs 9 and 10 in their 2009 patent application [2] but that the stated number of weeks post-infection in the figure legends and text are different (6 weeks in [1], 8 weeks in Fig 9A in [2] and 28 weeks in Figs 9B and 10 in [2]). The authors stated that these discrepancies are due to reporting errors in [2] and that the Fig 7 legend in [1] is correct.

## Supporting information

S1 File Western blot revealed with an anti-A2 mAb. A2 expression by 293 host cells infected with different clones of Adenovirus5-A2, and the appropriate negative control of a cell infected with an adenovirus5 expressing the CS protein from Plasmodium and non-infected cells, as indicated.
(PDF)
