# Supplementary figures and images for: Editorial Note: Clinical and Parasitological Protection in a Leishmania infantum-Macaque Model Vaccinated with Adenovirus and the Recombinant A2 Antigen
Source: PLoS Negl Trop Dis. 2024 Sep 12;18(9):e0012499. doi: 10.1371/journal.pntd.0012499 (PMC11392534; doi:10.1371/journal.pntd.0012499)

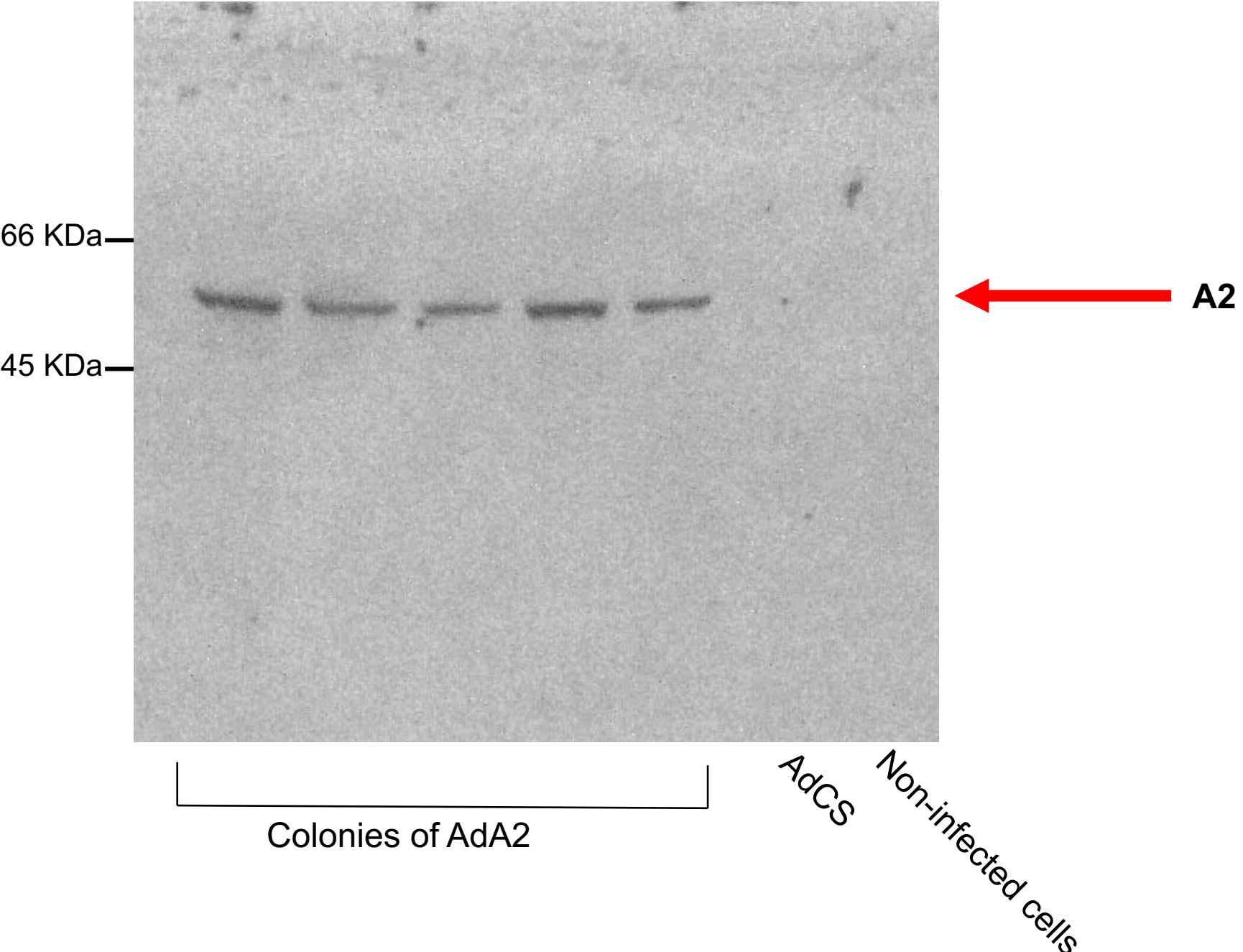

Supplement: S1 File — A2 expression by 293 host cells infected with different clones of Adenovirus5-A2, and the appropriate negative control of a cell infected with an adenovirus5 expressing the CS protein from Plasmodium and non-infected cells, as indicated. (PDF) [file pntd.0012499.s001.pdf]
